# Influence of support media supplementation to reduce the inhibition of anaerobic digestion by phenol and ammonia: Effect on degradation performances and microbial dynamics

**DOI:** 10.1016/j.dib.2018.06.071

**Published:** 2018-06-26

**Authors:** Simon Poirier, Olivier Chapleur

**Affiliations:** Hydrosystems and Bioprocesses Research Unit, Irstea, 1 rue Pierre-Gilles de Gennes, CS 10030, 92761 Antony Cedex, France

## Abstract

Data in this article provide detailed information on the microbial dynamics within digesters supplemented with different support media (two types of zeolites, two types of activated carbons, one type of chitosan, one control) in presence of different inhibitory conditions (control without inhibitor, 1.3 g/L of phenol and 19 g/L of total ammonia nitrogen). Data include the operational conditions and degradation performance measurements, as well as microbial community analysis, by 16S rRNA gene sequencing, at different time points for the different conditions (samples). Sequencing data were generated by using IonTorrent PGM sequencer. This data is associated with the research articles “Improving anaerobic digestion with support media: Mitigation of ammonia inhibition and effect on microbial communities?” (Poirier et al., 2017) [1] and “Support media can steer methanogenesis in presence of phenol through biotic and abiotic effects” (Poirier et al., 2018) [2]. The sequencing data have been deposited with links to BioProject accession number PRJNA450513, in the NCBI BioProject database (https://www.ncbi.nlm.nih.gov/sra/?term=PRJNA450513). Samples accession numbers go from SAMN08940368 to SAMN08940426.

**Specifications Table**

**Table:** 

Subject area	*Biology*
More specific subject area	*Microbial ecology of anaerobic digestion*
Type of data	*Table, figure, raw sequencing data*
How data was acquired	Gas production and composition were measured respectively by using a differential manometer (Digitron 2082P, Margam, UK) and a micro gas chromatography (CP4900, Varian, Palo Alto, USA). Volatile fatty acids concentrations were quantified by ionic chromatography coupled with conductometric detection (Dionex 120, ThermoFisher). DNA sequencing was carried out with Ion Torrent Personal Genome Machine.
Data format	Raw, analyzed
Experimental factors	Liquid samples were centrifuged (10,000*g*, 10 min). Pellets and supernatants were stored separately at − 20 °C before respectively DNA extraction with Powersoil™DNA isolation kit (Mobio Laboratories Inc. Carlsbad) and dilution for VFA analysis.
Experimental features	54 anaerobic batch digesters were carried out to evaluate in triplicate and in three different conditions (without inhibitor, in presence of 19 g/L of total ammonia nitrogen and in presence of 1.3 g/L of phenol) the influence of 5 support media on anaerobic digestion performances and microbial dynamics.
Data source location	*Antony, France*
Data accessibility	*Data are available in the article. The sequencing data have been deposited in the bioproject PRJNA450313, with the dataset identifier (TaxID) 1263854* in the NCBI BioProject database https://www.ncbi.nlm.nih.gov/sra/?term=PRJNA450513

**Value of the data**

• Those data provide a link between anaerobic digester performance (biogas production and volatile fatty acids accumulation), inhibition type (phenol or ammonia), and reduction of the inhibition by different support media and microbial community composition.
• Sequencing data can be used to understand the variation of microbial community composition, abundance and diversity in anaerobic digesters inhibited or not by phenol or ammonia and supplemented with different type of support media.
• Sequencing data can be used to identify micro-organisms characteristics of the different types of inhibition in the presence of support media. Accessibility to 16S rRNA sequence data and detailed associated metadata allows researchers to perform new analyses with their own research purposes.
• A wide number of conditions were tested (18) in triplicates and in similar experimental system, with the same inoculum and feeding, at the same time. A very important number of samples were sequenced, at different time points (59).

## 1 Data

**Fig. 1:**
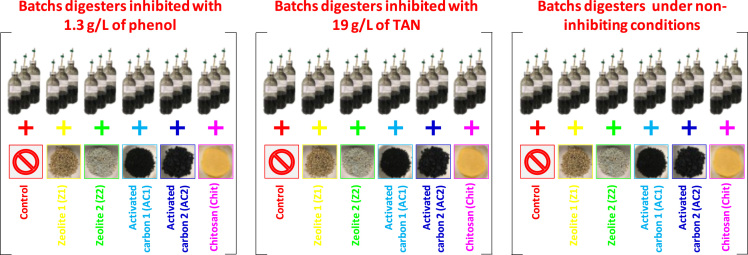
Experimental design. 54 anaerobic batch digesters were implemented to evaluate (in triplicate) the influence of five support media (zeolite no 1, zeolite no 2, activated carbon no 1, activated carbon no 2 and chitosan) on anaerobic digestion performances and microbial dynamics in three different conditions (in presence of 1.3 g/L of phenol, in presence of 19 g/L of total ammonia nitrogen and without inhibitor). Control digesters without support were also carried out for each condition.

**Fig. 2:**
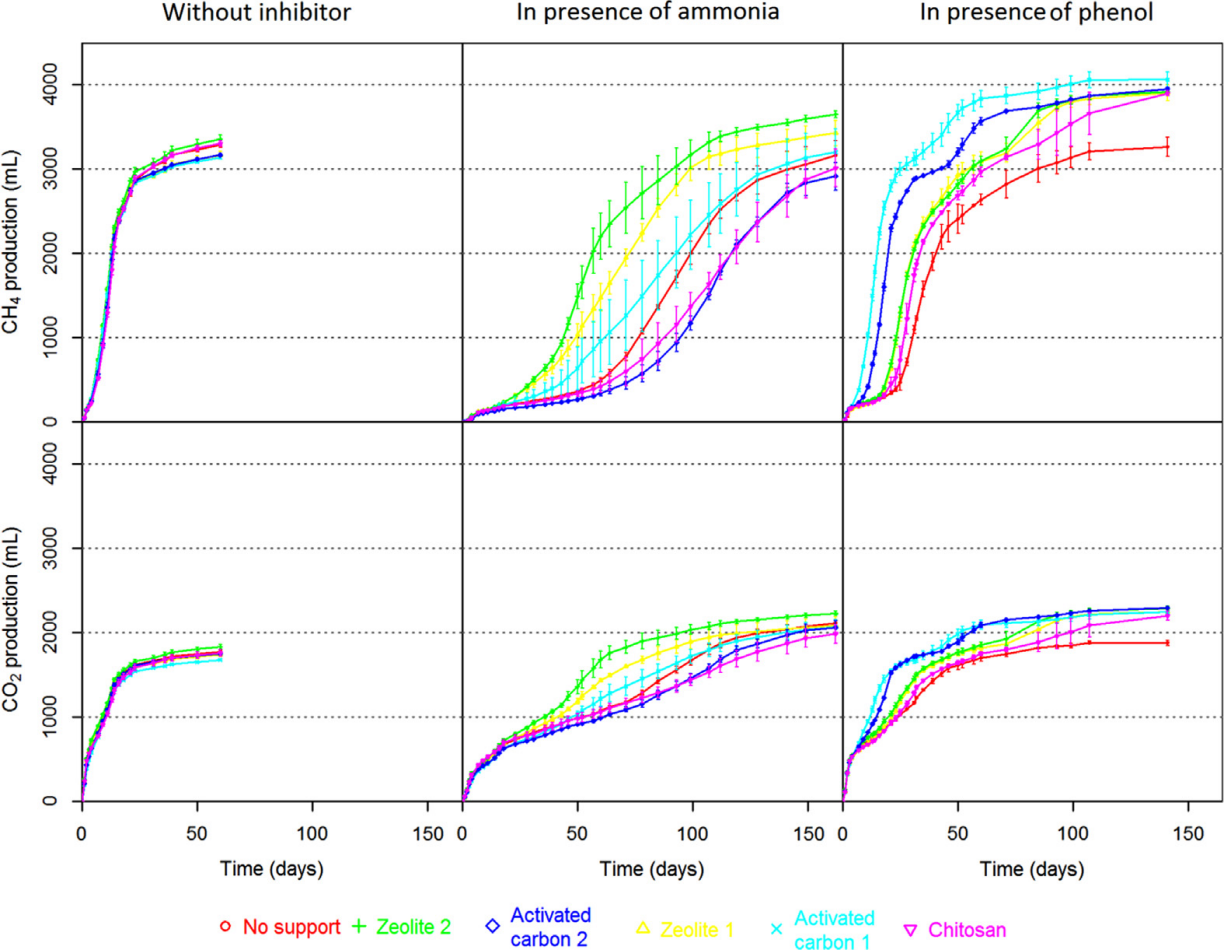
Cumulated CH_4_ and CO_2_ production (mL) over time (number of days) in presence of each support media initially added under the different inhibitory conditions. Mean values of each triplicate of bioreactors are presented for CH_4_ and CO_2_ production and error bars represent standard deviation within triplicates.

**Fig. 3:**
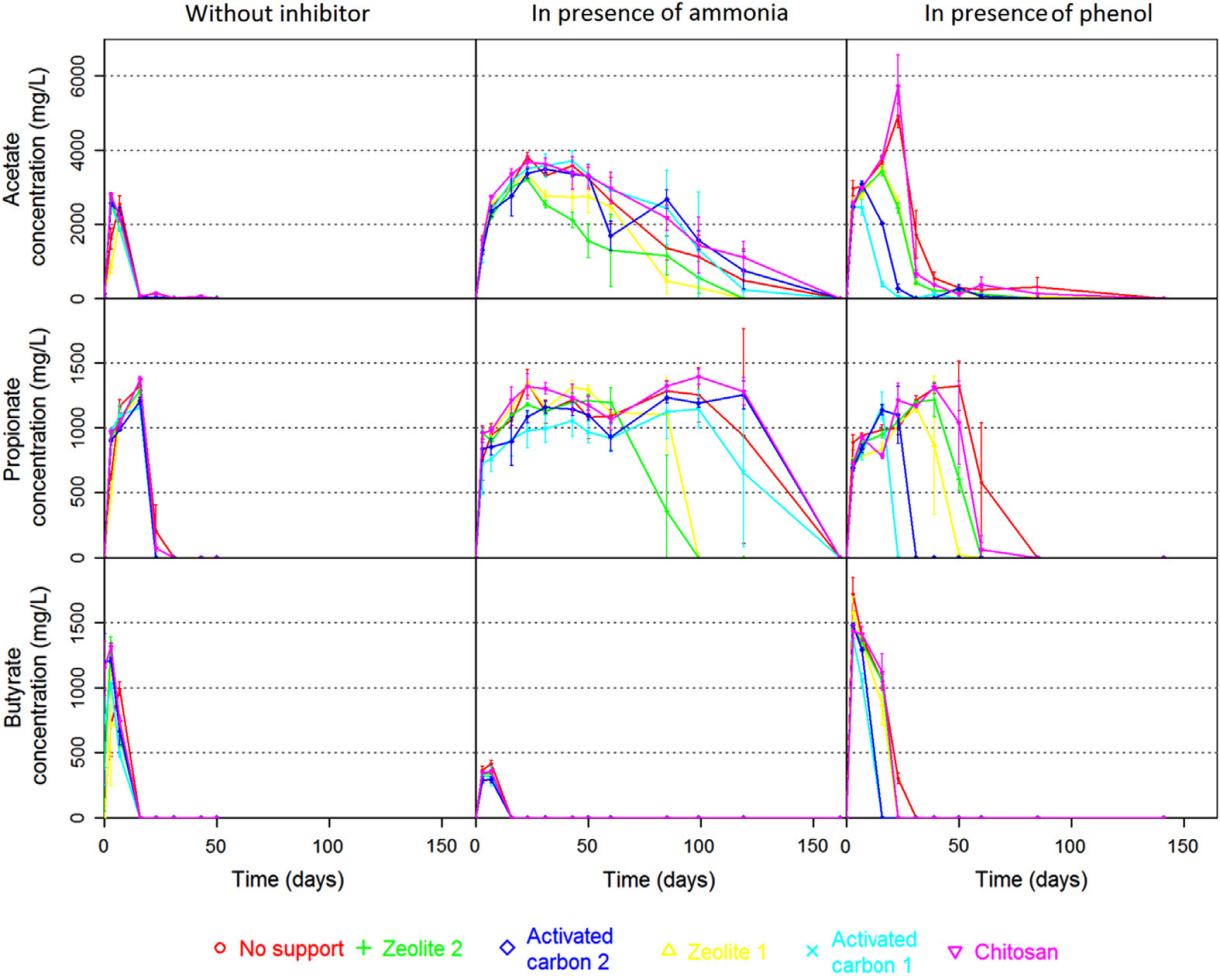
Volatile fatty acids concentrations (mg/L) over time (number of days) in presence of each support media initially added under the different inhibitory conditions. Acetate, propionate and butyrate concentrations in the liquid phase over time, for the different groups of triplicate bioreactors (mean values, error bars represent standard deviation within triplicates).

A wide variety of inhibitors can induce anaerobic digester disruption [3]. To avoid performance losses, support media such as zeolites [4], activated carbon [5] or chitosan [6] can be used to mitigate inhibitions. Fig. 1 illustrates the global experimental design of this study. Briefly, 54 anaerobic batch digesters were carried out to evaluate in triplicate and in three different conditions (without inhibitor, in presence of 19 g/L of total ammonia nitrogen and in presence of 1.3 g/L of phenol) the influence of 5 support media (2 zeolites, 2 activated carbons and chitosan) on anaerobic digestion performances and microbial dynamics. Data presented in Table 1 details support media introduced in each digester, the type of inhibitor (ammonia, phenol or none), as well as the samples which were selected to sequence the V4–V5 region of 16S rRNA. Table 2, Table 3, Table 4, Table 5, Table 6, Table 7 detail cumulated CH_4_ and CO_2_ production over time of each digester while Fig. 2 illustrates all these datasets (mean per triplicate of digesters). Similarly, Table 8, Table 9, Table 10, Table 11, Table 12, Table 13, Table 14, Table 15, Table 16 present the volatile fatty acids (acetate, propionate and butyrate) accumulation over time of each digester while Fig. 3 illustrates the same datasets (mean per triplicate of digesters).

**Table 1:** Sample nomenclature.

Inhibitor	Support media	Replicate	Bioreactor	Sampling time for 16S rRNA sequencing									
				Day 0 (T0)	Day 7 (T2)	Day 16 (T3)	Day 23 (T4)	Day 31 (T5)	Day 43 (T6)	Day 50 (T7)	Day 60 (T8)	Day 85 (T9)	Day 119 (T11)
None	None	No 1	None-1										
None	None	No 2	None-2	x		x							
None	None	No 3	None-3										
None	Zeolite No 1	No 1	Z1-1										
None	Zeolite No 1	No 2	Z1-2			x							
None	Zeolite No 1	No 3	Z1-3										
None	Zeolite No 2	No 1	Z2-1										
None	Zeolite No 2	No 2	Z2-2			x							
None	Zeolite No 2	No 3	Z2-3										
None	Activated carbon No 1	No 1	AC1-1										
None	Activated carbon No 1	No 2	AC1-2			x							
None	Activated carbon No 1	No 3	AC1-3										
None	Activated carbon No 2	No 1	AC2-1										
None	Activated carbon No 2	No 2	AC2-2			x							
None	Activated carbon No 2	No 3	AC2-3										
None	Chitosan	No 1	X-1										
None	Chitosan	No 2	X-2			x							
None	Chitosan	No 3	X-3										
Ammonia	None	No 1	NoneN1										
Ammonia	None	No 2	NoneN2	x	x		x		x		x	x	
Ammonia	None	No 3	NoneN3										
Ammonia	Zeolite No 1	No 1	Z1N1										
Ammonia	Zeolite No 1	No 2	Z1N2		x		x		x		x		
Ammonia	Zeolite No 1	No 3	Z1N3										
Ammonia	Zeolite No 2	No 1	Z2N1										
Ammonia	Zeolite No 2	No 2	Z2N2		x		x		x		x		
Ammonia	Zeolite No 2	No 3	Z2N3										
Ammonia	Activated carbon No 1	No 1	AC1N1										
Ammonia	Activated carbon No 1	No 2	AC1N2		x		x		x		x	x	
Ammonia	Activated carbon No 1	No 3	AC1N3										
Ammonia	Activated carbon No 2	No 1	AC2N1										
Ammonia	Activated carbon No 2	No 2	AC2N2		x		x		x		x	x	x
Ammonia	Activated carbon No 2	No 3	AC2N3										
Ammonia	Chitosan	No 1	XN1										
Ammonia	Chitosan	No 2	XN2		x		x		x		x		x
Ammonia	Chitosan	No 3	XN3										
Phenol	None	No 1	NonePhi1										
Phenol	None	No 2	NonePhi2	x	x		x	x		x			
Phenol	None	No 3	NonePhi3										
Phenol	Zeolite No 1	No 1	Z1Phi1										
Phenol	Zeolite No 1	No 2	Z1Phi2		x		x	x	x				
Phenol	Zeolite No 1	No 3	Z1Phi3										
Phenol	Zeolite No 2	No 1	Z2Phi1										
Phenol	Zeolite No 2	No 2	Z2Phi2		x		x	x	x				
Phenol	Zeolite No 2	No 3	Z2Phi3										
Phenol	Activated carbon No 1	No 1	AC1Phi1										
Phenol	Activated carbon No 1	No 2	AC1Phi2		x	x		x					
Phenol	Activated carbon No 1	No 3	AC1Phi3										
Phenol	Activated carbon No 2	No 1	AC2Phi1										
Phenol	Activated carbon No 2	No 2	AC2Phi2			x		x					
Phenol	Activated carbon No 2	No 3	AC2Phi3										
Phenol	Chitosan	No 1	XPhi1										
Phenol	Chitosan	No 2	XPhi2		x		x	x		x			
Phenol	Chitosan	No 3	XPhi3

**Table 2:** Cumulated CH_4_ production (mL) over time (days) for the different support media initially added under non-inhibiting condition.

CH4 production (mL)																		
**Sampling time (days)**	**None-1**	**None-2**	**None-3**	**Z1-1**	**Z1-2**	**Z1-3**	**Z2-1**	**Z2-2**	**Z2-3**	**AC1-1**	**AC1-2**	**AC1-3**	**AC2-1**	**AC2-2**	**AC2-3**	**X-1**	**X-2**	**X-3**
0	0	0	0	0	0	0	0	0	0	0	0	0	0	0	0	0	0	0
1	50.7	55.7	53.6	45	49	56.1	56.4	60.9	56.5	46.7	47.3	48.8	43	42.8	44.1	51.3	49.7	54.6
2	143.7	158.5	143.8	135.5	147	151.6	153.5	160.4	159	151.3	142.5	141.8	146.1	144.5	135.1	149.5	138.8	153
3	190.3	211.3	193.7	185.5	199.1	198.2	203.9	214.4	213.1	217	197.7	191.4	199.1	196.1	178.6	190.2	178	189.8
4	237.1	276.6	244.6	246.1	266.3	261.4	272.6	279	287.3	303.4	265.9	256	253.2	267.2	222.7	228.3	211.4	228.1
7	540.5	674.9	579	692.3	712.9	676.7	711.1	766.9	732.8	727.2	664.8	646.7	613.4	602.3	491.6	557.8	472.4	515.6
9	957.9	1147.9	1007.4	1117.7	1176	1130.9	1114.4	1173.8	1158.3	1151.9	1052.2	1047.8	1014.5	969.9	820.7	980.7	838	899.3
11	1407.1	1605.2	1454.8	1541.6	1614.5	1542.8	1527.4	1601.9	1597.4	1591.2	1468.6	1467	1460.4	1386.1	1225.1	1393.9	1205.7	1277.9
13	1924.4	2123.6	1986.7	2004.4	2093.7	2078.9	2041.4	2096.3	2119.6	2065.8	1966.2	1962.5	2035.1	1955	1797.2	1934.3	1704.4	1773
14	2149.1	2340.1	2206.6	2164.7	2303.2	2283.4	2274.7	2295.6	2359.4	2240.4	2181.1	2177.6	2260.2	2201.3	2069.5	2198.6	1974.9	2047.8
16	2388.3	2527.8	2406.7	2333.4	2433.4	2444.4	2483.5	2431.6	2561.1	2388.7	2384.5	2338.8	2425.6	2396.3	2327.5	2479.3	2324.2	2363.9
18	2565.2	2668.2	2541.8	2473.8	2589	2581.5	2603.9	2575.4	2685.9	2523.3	2517.8	2471.3	2576.1	2528.2	2486.1	2599.8	2486.1	2506.2
21	2664.8	2826	2679.6	2702.8	2847.9	2828.2	2840.7	2824.4	2924.2	2760.1	2747.2	2696.9	2814.4	2732.7	2697.4	2792.9	2678.5	2705.1
23	2812.5	2985.5	2819.9	2758.4	2913.1	2887	2966.1	2887.5	3058.6	2846.4	2858	2799.5	2897.2	2858.6	2829.9	2970.6	2850.9	2852
31	3019.3	3104.9	2994.2	2833.7	2992.9	2963.2	3069.6	2983.7	3171.1	2933.5	2948	2886.1	2983.7	2946.3	2930.3	3114	2992.4	3004
36	3075	3157.2	3046.4	2901.2	3056.7	3028.6	3147.2	3060.9	3251.8	2998.3	3012.9	2946.7	3039.6	2998.4	2989.2	3182.2	3072.5	3080
39	3161.4	3238.8	3117.7	2961.1	3113.7	3086.8	3211.8	3130	3323.2	3039.2	3054.6	2989.8	3080.4	3038.3	3036.2	3238	3125.1	3136.3
50	3207.7	3297.1	3186.8	3015.9	3180.6	3150.9	3289.6	3208.8	3399.5	3099.2	3113.4	3049.3	3149.8	3103.6	3101.8	3326.4	3202.6	3215.5
60	3271.9	3354	3240.1	3059.8	3238.5	3192.5	3348.8	3264.3	3456.6	3149.9	3165.6	3094.2	3206	3152.1	3147	3379.7	3256.5	3275.4

**Table 3:** Cumulated CH_4_ production (mL) over time (days) for the different support media initially added in presence of 19 g/L of Total Ammonia Nitrogen.

CH4 production (mL)																		
**Sampling time (days)**	**NoneN1**	**NoneN2**	**NoneN3**	**Z1N1**	**Z1N2**	**Z1N3**	**Z2N1**	**Z2N2**	**Z2N3**	**AC1N1**	**AC1N2**	**AC1N3**	**AC2N1**	**AC2N2**	**AC2N3**	**XN1**	**XN2**	**XN3**
0	0	0	0	0	0	0	0	0	0	0	0	0	0	0	0	0	0	0
1	0.6	0.6	0.6	0.7	1.3	0.6	0.7	1.3	1.3	0	0	0	0	0	0	0.7	0	0.6
2	19.1	19.8	17.9	15	13.9	10.1	11.8	15.3	19.2	9.2	6.5	8.8	6.5	5.5	5.9	8.7	3.1	8.4
3	38	36.4	36.4	36.5	42.7	33.7	36.3	40.4	45.7	30.8	9.7	34.2	15.7	20.7	21.2	30	10.7	28.2
4	79.3	78.1	72.2	76.5	89.6	72.3	71.3	76.9	86.3	56.6	18.7	69.2	32.1	41.6	39.4	66.7	24	59.1
7	119.6	120.6	118.8	124.4	127.6	114.1	115	112.6	121.3	102.6	83.1	121.6	94.7	101.4	96	127.3	103.1	118.9
9	133.2	132.9	131	138.4	139.7	127.3	130.1	128.3	136.6	114.4	96	134.8	104.8	109.1	105.4	141	116.6	130.1
11	144.9	148.3	144.5	152.1	155.6	141.7	147.2	142.7	152.2	126.9	105.6	149.1	115.6	121.1	115.1	153.5	129.5	144.1
14	163	165	161.7	183.2	179.9	170.8	184.8	171.6	186.4	154.3	124.6	179.9	131.8	133.4	126.3	170	147.4	159.7
16	178	180.4	177.7	213.9	211	194.3	210.2	194.8	210.6	188.5	146.8	211.8	147.2	149.9	143.3	187.2	161.9	172.9
18	198.2	199.6	196.9	245.7	246.4	220.2	240.5	225.2	239.5	208.6	161.7	237.6	158.8	161.4	156.9	202.5	177.5	187.8
23	219.3	220.5	217.7	307.7	335.6	277.7	320.2	298.2	311.6	244.7	190.3	293.7	174.1	173.9	171.9	220.8	198.1	204.1
28	239.2	240.3	237.8	387.7	450.9	362.6	447.3	401.1	423.9	275.1	217.7	352.2	188	186.5	185.4	234.4	216.9	218.1
31	256.5	255	255	442.9	529.3	422.2	539.7	478.2	507.9	293.4	234.1	387.3	196.4	194	193.6	242.6	228.3	226.5
36	277.9	275	277.1	531.7	649.6	521	684.5	603.5	641.5	341.2	272.6	478.7	215.7	208.9	212.7	266.5	259.4	246.2
39	296.5	290	292.2	597.2	737.5	604.3	786	707.2	754.6	369.9	295.7	533.5	227.3	217.8	224.1	280.8	278	258.1
43	319	310.9	314.2	687.3	856.6	747.8	946.2	894.8	963.3	419.3	330.2	636.9	242.6	229.8	239.1	298.2	306.7	273.6
46	344.7	332.3	334.8	771.5	954.9	906.7	1125.4	1122.7	1250.9	471.5	375	754.4	256.7	253.2	249.9	323.2	332.7	287.4
50	375.9	357	359.7	886.3	1083.8	1135.1	1364	1447.6	1649.6	556.1	436	920.5	276.2	262.6	267.3	334.8	375.2	308
52	402	378.3	380.5	970.8	1186.3	1280.5	1470.7	1639.3	1858.7	619.8	532.1	1027	291.9	269.8	280.1	347.7	399.5	316
57	467.4	431.6	432.5	1180.2	1348.6	1507	1762.5	2037.7	2292.7	737	623.9	1237.9	331.2	287.8	312	380.2	460.4	336.1
60	527.3	483.9	486.1	1310.5	1490.5	1635.6	1924.2	2210.8	2475.5	823.3	689	1376.2	368.5	303.2	357.6	405	504.8	358.9
64	625.7	562.8	559.7	1502.1	1687.2	1763.2	2071	2351.9	2619	939.4	760.1	1497.4	419.9	328	396.9	445.1	597.2	398.2
71	828.4	758.5	739.2	1837.5	2031.3	1986.6	2259	2501.7	2863	1155.9	915.8	1723.8	502.8	382.1	507.7	532.9	796.7	472.4
78	1109.8	1078.4	1042.5	2242.2	2286.2	2208.4	2437.8	2666.1	3053	1422.5	1114.2	1950	622.2	459.9	640.8	647.7	1019.4	584.6
85	1365	1393.1	1346.8	2646.8	2541.1	2430.3	2606	2809.6	3178.2	1729.6	1332.2	2158.3	772.9	596	808.8	814.7	1216.1	755.7
93	1642.2	1790.9	1736.3	2945.9	2813.6	2679.3	2869.1	2956.8	3279.7	2089	1559	2382.2	995.3	822.3	1017.7	1064.9	1400.9	1012.9
99	1853.2	2110.6	2055.6	3170.1	3017.9	2866	3069.6	3079.2	3367.4	2348.4	1761.9	2555	1231.6	1076.4	1214.3	1308.5	1558.8	1243.8
107	2280.2	2490.8	2277.7	3316.3	3111.3	3017.1	3275.8	3242.3	3452.8	2573.3	2033.2	2768.8	1575.8	1450.1	1517	1666.5	1751.5	1498.5
112	2444.2	2647.4	2477.5	3347.8	3141.5	3067.6	3357.9	3346.8	3458.4	2671.4	2205.6	2897	1830.6	1746.2	1786.2	1934.5	1904.2	1665.7
119	2731.9	2727.9	2615.5	3393.9	3189.3	3119.4	3415.3	3417.6	3497.3	2837.7	2409.1	3032	2127.3	2054.1	2140.2	2258.3	2116.2	1860.5
128	3054.9	2812.1	2738.8	3439.5	3239.1	3165.7	3474.1	3479.6	3534	3053.4	2586.2	3170.9	2338.5	2348.5	2440.4	2616.8	2333.4	2152.4
141	3241.5	2852.1	2896.3	3488.9	3293.7	3227.1	3530.2	3536.3	3593.4	3228.4	2730.1	3240.8	2698	2622.6	2839.6	2971	2546.6	2525.7
149	3286.7	2893.8	2987.9	3529.2	3331.4	3261.5	3567.4	3576.2	3640	3308.9	2799.2	3293.4	2816	2693.2	3007.2	3090.3	2652.7	2887.9
162	3351.4	3009.9	3138.1	3598.3	3380.2	3309.2	3623.4	3632.9	3694.3	3373	2885.3	3350	2893.7	2764.1	3087.5	3193.2	2761.9	3084

**Table 4:** Cumulated CH_4_ production (mL) over time (days) for the different support media initially added in presence of 1.3 g/L of phenol.

CH4 production (mL)																		
**Sampling time (days)**	**NonePhi1**	**NonePhi2**	**NonePhi3**	**Z1Phi1**	**Z1Phi2**	**Z1Phi3**	**Z2Phi1**	**Z2Phi2**	**Z2Phi3**	**AC1Phi1**	**AC1Phi2**	**AC1Phi3**	**AC2Phi1**	**AC2Phi2**	**AC2Phi3**	**XPhi1**	**XPhi2**	**XPhi3**
0	0	0	0	0	0	0	0	0	0	0	0	0	0	0	0	0	0	0
1	19.9	21.8	10.4	20.8	18.2	22.4	25.3	25.7	26	30.3	21.8	22.8	28.5	24.4	22.8	15.6	23.5	24.8
2	109.1	100.9	64.8	102.5	91.4	91	99.1	102	102.5	126.9	111.4	107.1	115.5	110	102	90.6	99.9	103.1
3	167	157.4	139.9	149.5	144.6	138.8	161.8	157.3	159.4	165.9	159.1	155.1	159.9	157.5	149.4	155.4	159.7	159.4
4	179.1	169.6	151.7	157.9	154.7	148.9	174.1	170.1	173.5	193.1	189.7	188.9	174.4	179.8	169.5	170.2	172.2	172.7
7	206	195.5	180.6	191.3	184.6	174.6	204.4	203.9	199.7	373.1	391.9	368.7	228.8	229.4	229.1	194.2	196.4	195.6
9	227.6	211	203.6	215	211.1	188.5	235.3	232.1	224.7	668.8	663.5	652.7	298.5	311.3	287	209.1	209.6	211
11	238.5	226.4	212.2	226.6	219.8	199	254.8	246.6	241.7	1062.5	1027.5	1002.6	408.3	445.3	407.3	218.2	220.1	221.8
13	259	242.2	227.6	252.3	241.9	227.2	277.7	266.4	263.3	1567.3	1510.3	1430.1	682.6	710.6	661.1	233.5	236.8	243.3
14	269.2	250.1	235.3	265.1	252.9	241.4	289.2	276.3	274.1	1847.6	1797.1	1700.1	819.8	843.3	788	241.2	245.2	254
16	276.9	269.1	273.9	307.9	288	285.1	332.4	315.1	311.2	2298.7	2257	2164	1161.7	1176.8	1132.9	263.5	271.2	294
18	342	300.6	290.7	394	360.4	366.3	432.9	404.2	392.1	2617.4	2530.1	2457.7	1626.7	1623.7	1550.3	295.8	307.7	345
21	459.9	354	338.4	672.9	607	653.4	734.6	692.7	653.5	2875.3	2743.8	2774.5	2328.9	2314.3	2248.4	393.9	431.5	540.7
23	598.1	403.9	382.1	967.3	874.8	960	1014.3	986.6	907.2	3020.8	2885.7	2862.9	2457.7	2450.4	2398	442.6	496.7	638
25	838.8	490.2	448.5	1321.5	1220.8	1225.3	1342	1329.8	1225.7	3084.2	2978.2	2937.1	2613.5	2610.9	2577.6	589.3	695.2	911.9
28	1311	778.4	644.2	1794.1	1710	1719.7	1758.2	1745.6	1682.4	3149	3040.8	2996	2744.2	2748.4	2730.9	1056.3	1201.3	1411.5
31	1767.4	1127.3	1066.2	2134.8	2061.7	2048	2068.4	2040.3	2030.7	3208.4	3095.5	3047.3	2875	2886	2884.2	1661.9	1742.1	1824.2
32	1881.8	1262.5	1194.2	2225.9	2151.7	2133.1	2159.3	2126.8	2126.3	3236.6	3120.7	3066	2885.8	2897	2895.1	1808.5	1875.8	1929.8
35	2167.7	1596	1557.2	2433.3	2353.7	2301.5	2350.6	2306.4	2320.2	3321.1	3196.5	3122.2	2918.2	2930.2	2927.9	2109.1	2151.4	2149.3
39	2418.9	1838.1	1967.5	2614.3	2525.4	2446.4	2520.9	2468.5	2497.4	3433.9	3297.5	3197.2	2961.4	2974.4	2971.5	2344.9	2346.8	2354
43	2595	2124.2	2272.3	2766.6	2651	2570.5	2638.4	2579.8	2609.6	3546.6	3398.5	3272.2	3004.5	3018.6	3015.2	2497.7	2480.5	2491.7
46	2679.4	2219.4	2425.4	2916.7	2756.5	2686.6	2728.5	2655	2685.7	3653.7	3543.4	3427.7	3108.1	3073.5	3100.3	2592.9	2575	2602.3
50	2755	2280	2540.4	3047.5	2923	2818.2	2873	2775.3	2813.7	3768.4	3663.4	3586.3	3246.1	3146.6	3213.9	2670.3	2648.6	2741.9
52	2819.6	2329	2584.4	3077.9	2961.9	2848.6	2937	2836.3	2876.7	3822	3707	3638.4	3347.5	3216.6	3315	2720.7	2697.1	2797.7
57	2981.1	2451.4	2694.2	3153.9	3059.3	2924.6	3097	2988.8	3034.4	3901.2	3767.5	3707	3518.2	3416.7	3519.3	2846.9	2818.4	2937.3
60	3041.6	2559.4	2715	3195.8	3105	2962.9	3152.4	3046.6	3095.4	3941.3	3812.1	3753.5	3593.5	3517.1	3595	2961.4	2916.6	3027.2
71	3244.9	2825.3	2815.3	3298.9	3195.9	3079.8	3390.5	3150.5	3197.7	3978	3848.5	3792	3695.9	3695.7	3674.9	3182.2	3118.2	3130.5
85	3480.6	2964.2	3053.3	3522.4	3660.6	3468.4	3784.3	3612.9	3696.3	4024.7	3894.9	3841	3739.9	3751	3721.7	3500.8	3183.6	3197.1
93	3725.9	2961.3	3195.1	3762.4	3747.9	3680.3	3836.5	3731.6	3751.6	4074.1	3941	3886.9	3790.3	3798.6	3766	3741.2	3301	3255.1
99	3767.6	3024.5	3254.1	3865.3	3792.1	3731.4	3879.5	3785.7	3792.1	4117.2	3982	3931.1	3833.7	3837.8	3802.6	3823.9	3490.3	3297
107	3823.7	3109.7	3303.8	3914.5	3834.7	3764.5	3918.6	3835.4	3847.8	4162.6	4031.6	3977.1	3879.7	3879.4	3851.1	3865.4	3732.6	3383.1
141	3879.8	3176.8	3351.5	3999.8	3894.2	3817.4	3973.2	3878.5	3901.7	4239.9	4118.3	4062.9	3955.9	3969.7	3926.7	3933.7	3880.1	3884.5

**Table 5:** Cumulated CO_2_ production (mL) over time (days) for the different support media initially added under non-inhibiting condition.

CO2 production (mL)																		
**Sampling time (days)**	**None-1**	**None-2**	**None-3**	**Z1-1**	**Z1-2**	**Z1-3**	**Z2-1**	**Z2-2**	**Z2-3**	**AC1-1**	**AC1-2**	**AC1-3**	**AC2-1**	**AC2-2**	**AC2-3**	**X-1**	**X-2**	**X-3**
0	0	0	0	0	0	0	0	0	0	0	0	0	0	0	0	0	0	0
1	217.6	274.5	248	257.5	269.6	260.4	247.1	252.4	293.4	187.7	202.7	201.7	226.6	207.9	207.2	255.7	236	243.5
2	449.2	510	476.1	464.3	500.4	485.5	497.1	496.9	526.9	396.2	393.3	389.6	446.4	436.2	424.9	497.4	468.7	479.6
3	573.7	631.4	596.7	569.8	618	599.8	616.1	604.7	633.9	498.4	497.4	492.6	546.3	539.6	519.1	589.1	559.7	568.4
4	665.7	745.6	689.2	654.4	725.8	714.6	731.9	700.3	752.9	594.8	586.5	566.4	617.4	654.4	609.1	653.9	619.2	630.1
7	830.4	915	855.6	833.6	897	880.1	892.8	879.7	918	775.4	767.5	754.3	821.8	822.9	775.5	808.1	755.5	764.4
9	963.3	1055.6	988.9	970.1	1040.1	1014.4	1023.7	1021.5	1059.5	940.7	918.1	910.4	978.5	966.1	911.9	960.5	880.5	894.8
11	1091	1190.4	1121.1	1101.9	1169.7	1144.3	1152	1157.1	1195.2	1080.7	1047.3	1040.7	1120.5	1096.8	1039.2	1102.2	999.8	1015.2
13	1257.8	1364.1	1290.5	1262.5	1335.9	1319.1	1325.1	1326.6	1371.4	1246.6	1221.5	1209.6	1309.9	1279.9	1214.1	1281.1	1159.8	1178.2
14	1361.8	1469.3	1394.5	1346.7	1436.6	1418.6	1436.1	1427.7	1482.2	1333.1	1324.8	1311.8	1418.7	1394.6	1334.7	1398.9	1260.2	1280.5
16	1435.1	1529	1456.9	1412.4	1473.8	1480.3	1506.8	1480.6	1550.4	1392	1392.6	1366.3	1480.3	1459.2	1417.6	1499.7	1360.7	1373
18	1514.3	1595.7	1516.3	1463.5	1538.6	1534.1	1561.8	1530.3	1605.4	1445.8	1454	1424.7	1541.1	1518	1484.8	1568.4	1434.8	1437.1
21	1545.9	1634.8	1557	1508.9	1595.9	1586.5	1615.5	1581.7	1662.6	1506.1	1514	1483	1597.1	1569.7	1543.5	1627.3	1496.2	1492.8
23	1591	1682.3	1600.9	1544.6	1635.9	1623.6	1661.7	1619.8	1709	1548.1	1557.9	1525.6	1639.2	1612.9	1590.6	1685.1	1549.7	1542.6
31	1638.2	1722.3	1645.2	1578.2	1671	1658.3	1704.5	1658.1	1756.2	1593.1	1599.3	1566.2	1684.9	1654.3	1635.7	1735.2	1598.9	1590.5
36	1668.4	1747.1	1673	1609.7	1703.6	1689.1	1743	1697.2	1796.9	1615.7	1624	1586.5	1705.6	1675.2	1662.8	1769.7	1640.9	1631.7
39	1694.7	1777.9	1695.8	1630	1721.5	1710.5	1767.8	1724.9	1825.8	1637.7	1644.9	1605.8	1726.4	1693.1	1683.5	1794	1663.5	1655.1
50	1721.3	1803.5	1729.2	1656.1	1750.4	1737	1803.5	1760.6	1858.6	1663.5	1671.6	1631.7	1759.6	1723.3	1713.8	1828.8	1696.1	1687.6
60	1747.9	1829.2	1753	1682.4	1779.2	1760.8	1832.4	1788.7	1887.4	1687	1695.4	1654.2	1785.6	1746.8	1735.6	1855.5	1720.3	1714

**Table 6:** Cumulated CO_2_ production (mL) over time (days) for the different support media initially added in presence of 19 g/L of Total Ammonia Nitrogen.

CO2 production (mL)																		
**Sampling time (days)**	**NoneN1**	**NoneN2**	**NoneN3**	**Z1N1**	**Z1N2**	**Z1N3**	**Z2N1**	**Z2N2**	**Z2N3**	**AC1N1**	**AC1N2**	**AC1N3**	**AC2N1**	**AC2N2**	**AC2N3**	**XN1**	**XN2**	**XN3**
0	0	0	0	0	0	0	0	0	0	0	0	0	0	0	0	0	0	0
1	43.9	48.3	44.6	45.4	45.5	45.5	47.3	49.5	57.6	43.6	41.3	48.2	52.6	54.6	54.1	61.7	58.3	56.4
2	136.5	136.1	124.7	127	132.9	122	127.2	136.4	156.4	109.6	96.5	109.8	111.4	117.8	113.1	142.7	124.1	134
3	225.2	236.3	224.8	229.8	241.7	223.8	234.7	243.1	264.1	199.7	161.8	204.1	199.6	212.7	205.5	248.6	209.7	232.3
4	308.8	322.1	304.7	311.8	334.6	304.7	316.7	324.4	350.3	263.8	216.9	277.6	263.2	281.2	271	338.2	274.3	311
7	401.1	419.2	403.6	412	419.8	395.6	412.5	412.6	434.7	358.9	354.3	374.9	380.8	389.4	386.5	453.5	420.9	424.8
9	447	469.5	452.6	464.1	463.8	440	466.4	474	483.2	405.7	401	418.9	415.8	425.2	432.2	508.5	480	473.5
11	486.7	510.7	493.1	504.7	501.8	479.3	510	502.8	528.2	443.1	435.5	453.5	451.2	457.1	462.3	550.7	516	517.3
14	559.9	582.9	563.5	585.1	565.9	564.4	609.6	575.5	612.7	517	503.7	522.1	513.3	517.5	528.1	616.6	572.9	572.9
16	606.5	632.5	612.7	655.2	620.2	615.1	670.6	632.9	674.7	597.2	568.7	584.8	572.8	573.3	579.4	668.7	616.2	613.3
18	666.5	693.9	671.4	717.8	678.7	666.9	732	698.1	736.3	644	614	636	624.8	625	632	734.1	674.9	673.9
23	726.8	757.6	730.8	787.7	754	725.6	812.4	780.7	810.2	715.1	677.7	701.3	681	677.1	690.6	793.1	729.5	721.3
28	774.8	808.7	778.2	852.7	821.5	785.6	888	856.6	881.8	756	719.9	744.8	717.5	713.6	727.9	830.3	764.7	754.8
31	812.9	845.2	815.7	902.7	876.4	831.4	951	912.6	940.5	780.5	745.2	770.9	739.3	735.5	750.2	852.7	785.8	774.9
36	852.9	886.1	858.5	960.1	942	888.2	1025.2	985	1012.1	839.9	807.8	840.7	791	782.9	802.1	910.3	837.6	824.9
39	882.6	912.3	883.3	1006.2	993.9	935.8	1084.6	1046	1078	875.5	845.4	882.5	822.1	811.4	833.2	945	868.8	854.9
43	910.4	942	911.7	1052.9	1049	989.8	1150.6	1112.2	1157.5	923.3	897.7	938.7	856.6	842.4	870.6	980.4	905.6	885.6
46	954.3	983.6	951.9	1105.4	1113.5	1077.4	1234.9	1203.4	1312.8	976.1	956.4	1007.5	896.1	869.7	900.3	1016.7	942.5	922
50	983.6	1009.5	977.2	1154.1	1221.3	1198.9	1318.9	1302.7	1458.8	1026.4	1008.9	1075	915.7	901.4	932.1	1046.7	974.6	935.4
52	997.9	1023.1	991.3	1206.1	1290.1	1280	1372.7	1405.2	1561.6	1078.4	1034.5	1130.7	928.7	911.9	943.9	1058.5	992.2	948.1
57	1033.8	1057.2	1026.5	1335.5	1360.8	1377.1	1465.9	1592.6	1687.4	1127.4	1103.3	1238.7	961.4	938	973.6	1088	1036.3	979.7
60	1071.9	1089.3	1065.2	1419.2	1435.5	1459.8	1580.4	1694.7	1777	1180.4	1154.7	1317.6	1002.3	972.6	1002.2	1120.4	1052.8	1013.7
64	1121.3	1140	1110.8	1492.4	1494.4	1505.9	1681	1763.6	1838.9	1240.1	1210	1402.5	1049.9	1010.4	1058.4	1167.5	1107.2	1060.1
71	1174.4	1189.5	1159	1620.5	1597.4	1586.7	1758.4	1825.5	1913.2	1326.3	1280.6	1493.2	1100.9	1059.3	1116.8	1219.7	1173.6	1108.4
78	1269	1323.7	1281	1733.5	1669.1	1642.2	1821.8	1881.9	2001.9	1432.8	1370.1	1573.2	1171.3	1110.6	1191.9	1283.9	1249.6	1164.4
85	1422.1	1469.4	1406.2	1846.5	1740.7	1697.8	1868.6	1927.8	2033.8	1542.9	1459.4	1633.5	1276.2	1207.6	1308.4	1338.4	1324.1	1217.2
93	1540.7	1608.1	1534.3	1932.5	1811.4	1773.7	1932.6	1975.8	2071.3	1687.8	1550.9	1690.3	1372.1	1312.4	1419.9	1412.7	1409.5	1288.8
99	1630	1724.9	1651.9	1997	1864.5	1830.6	1998.5	2012.6	2102.1	1785.4	1629.1	1746.1	1467.4	1406	1512.1	1485.5	1477.8	1367.2
107	1764.6	1871.4	1772.4	2059.1	1915.9	1869.3	2052.5	2057	2129	1868.1	1710.1	1813.8	1590.6	1535.5	1628.4	1596.3	1554.6	1461.9
112	1855.1	1944.8	1838	2090.7	1940.2	1898.2	2091.7	2091.6	2146.6	1919.4	1770.5	1858.8	1692.3	1651.7	1733.4	1694.5	1610.5	1532.5
119	1938.1	1995.7	1893.3	2106.5	1958	1916	2119.2	2117.7	2163.5	1975.9	1829.1	1903.8	1787.5	1749.4	1844.7	1801.4	1669.3	1607.8
128	2016.3	2030.6	1939.3	2129	1977.3	1938.4	2144.8	2143.4	2181.9	2032.3	1881.5	1945.1	1855.8	1829.6	1929.2	1907.4	1726.6	1692.8
141	2084.8	2060.6	1994.5	2159.6	2010	1973.2	2177.7	2174.3	2215.3	2100	1942.8	1992.5	1953.2	1922.4	2045.7	2016.1	1811.9	1794.2
149	2122.1	2082.6	2031.1	2175.3	2024.9	1988.2	2194.9	2193.2	2236.6	2130.9	1968.8	2007.7	2004.3	1967.4	2111	2066.1	1858.1	1891.5
162	2150.9	2116.3	2076.6	2206.2	2044	2010.4	2220.8	2218.9	2259.5	2166	2003.2	2034.1	2045.8	1994.4	2151.8	2110.6	1901.7	1950

**Table 7:** Cumulated CO_2_ production (mL) over time (days) for the different support media initially added in presence of 1.3 g/L of phenol.

CO2 production (mL)																		
**Sampling time (days)**	**NonePhi1**	**NonePhi2**	**NonePhi3**	**Z1Phi1**	**Z1Phi2**	**Z1Phi3**	**Z2Phi1**	**Z2Phi2**	**Z2Phi3**	**AC1Phi1**	**AC1Phi2**	**AC1Phi3**	**AC2Phi1**	**AC2Phi2**	**AC2Phi3**	**XPhi1**	**XPhi2**	**XPhi3**
0	0	0	0	0	0	0	0	0	0	0	0	0	0	0	0	0	0	0
1	109.6	122.6	110.4	116.7	108.4	129.1	141.4	130.9	151.5	124.5	113.8	105.3	121.1	123.7	114.9	104.7	116.9	119.7
2	331.6	341.7	301.4	327.5	310.8	322.2	346.6	334.7	360.7	348.5	326.7	311.2	349.5	337.7	329.1	332	335.7	335.3
3	492.4	488.4	479.9	470.9	455.9	457.1	497.9	480.6	500.6	459	447.6	431.9	467.1	466.5	455.6	491.1	487.4	475.2
4	542	537.1	533	531.1	511.8	510.2	553	536.9	551.6	533	520.9	522.1	540.2	534.1	526.8	539.4	532.9	521.9
7	626.7	616.3	626.8	631	604.7	593	643.8	638.4	638.3	705.7	689.2	680.6	654.6	635.3	658	617.3	615.6	599.5
9	685.6	660.4	696.5	699.6	682.5	633.7	722.8	711.9	703.3	846.7	825.5	818.9	740.6	740.7	730.4	662.7	649.5	641.3
11	710.5	701.9	719	721.5	698.4	654.6	775.1	753.7	752.6	972.6	949.9	930.8	817.2	832.4	820.2	688.6	678.7	672.1
13	750.3	735.5	756.2	761.8	735.7	699.9	812.3	788.1	787.3	1129.7	1100.2	1052.1	914.7	920.4	918.6	721.7	712	708.8
14	770.3	752.3	774.9	782	754.3	722.6	830.9	805.2	804.6	1242.5	1212.6	1163	963.4	964.5	967.8	738.2	728.7	727.1
16	822.1	799.4	827.7	840.6	806	778.2	892.7	861	853.8	1380.2	1350.1	1300.6	1092.1	1091.9	1088.2	783.4	774.1	792.8
18	896	856.1	890.3	913.7	875.6	846.1	976.4	944.5	932.1	1492.5	1454	1408	1238.3	1232.2	1214.3	833.4	824.6	852.9
21	983.6	909.7	940.9	1004.4	960.9	961.3	1068.7	1042.4	1018.3	1576.8	1530.6	1534.6	1547.9	1528.3	1507.9	921.6	913.6	968.3
23	1058.5	958.1	989.7	1110.5	1053.2	1080.8	1167	1138.9	1107.8	1636.5	1587	1568.5	1599.8	1584.8	1570.3	966.4	962.7	1023.8
25	1152.2	1016.4	1039.2	1209.8	1155	1183.2	1257.4	1228.3	1197.7	1667.7	1618	1591.9	1638.9	1629.8	1620.1	1032.7	1031.2	1098.8
28	1254.3	1096.5	1099.2	1337.1	1288.2	1304.7	1372.7	1340	1321.6	1697.1	1649.7	1617.6	1682.3	1676.9	1668.3	1134.2	1138.4	1196.1
31	1380.5	1156.4	1184.7	1462.8	1419.7	1423.9	1483.7	1444.1	1446.7	1726.4	1676.5	1641.6	1725.7	1723.9	1716.6	1267.9	1264.4	1314.8
32	1448.8	1216.3	1247	1519.7	1480.3	1476.9	1535.4	1494.3	1506.1	1741.4	1690.3	1652.3	1730.8	1728.9	1721.7	1339.8	1338.2	1380.4
35	1527.6	1305.3	1342.5	1586.4	1550.6	1534.1	1601	1555.2	1576.5	1786.5	1731.7	1684.2	1745.8	1743.9	1737.2	1436.1	1426.4	1449.6
39	1607.4	1385	1471.2	1651.1	1614	1588.3	1663.5	1616.4	1641.9	1846.7	1786.9	1726.8	1765.9	1764	1757.9	1526.3	1505.2	1522.2
43	1672.6	1468.9	1579.3	1707	1662.4	1637.9	1709.1	1662	1686.1	1906.8	1842.1	1769.4	1786	1784.1	1778.5	1586	1558.1	1574.9
46	1712.1	1514.8	1647.8	1761	1706.7	1685	1751.4	1697.9	1722.1	1975.2	1925.4	1859	1845	1814.4	1826.3	1622.9	1591.6	1614.7
50	1742.5	1539.7	1694.5	1805.6	1758.9	1726.2	1797.8	1740	1766.1	2038.2	1991.5	1933.9	1923.6	1854.9	1890.1	1652.4	1621	1666
52	1762.4	1559	1707.8	1816.6	1771.4	1736.4	1815	1757.4	1784.1	2079.2	2029.2	1975.3	1984.2	1902.2	1953.6	1672.4	1638.9	1683.8
57	1812.2	1607.2	1741.3	1844.1	1802.6	1761.8	1857.8	1801	1829.1	2124.8	2064.5	2016.7	2063.5	1993.2	2052	1722.5	1683.7	1728.4
60	1839.2	1645.6	1762.1	1866.1	1826.8	1781.9	1889.5	1828.9	1860.1	2154.1	2096.7	2046.3	2113.9	2056.3	2103.4	1768.4	1726.3	1767.9
71	1884.4	1706.3	1791	1909.5	1863.5	1835.6	2025.3	1869.3	1898.5	2170.7	2112.9	2064.6	2169.4	2145.3	2150.9	1828.1	1781.5	1802.4
85	2034.4	1817	1833.8	2016.4	2089.9	2015.9	2186.3	2086.9	2138	2191.8	2133.4	2087.9	2198.9	2186.1	2176.6	2021.3	1815.5	1833.3
93	2143.4	1815.8	1867.5	2143.3	2160.9	2149.5	2246.2	2180.7	2208.1	2218.1	2159	2112.5	2226.2	2211	2200.8	2141.5	1898.2	1855
99	2185.3	1815.5	1888.5	2209.8	2183.1	2174.8	2267.8	2208	2229.6	2241.7	2181.5	2136.1	2249.4	2231.6	2220.3	2180.4	1981.2	1870.6
107	2222.5	1862.8	1906.9	2246.1	2218.6	2205.1	2295.2	2241.3	2256	2269.7	2209.4	2164	2278.2	2259.7	2247.4	2228.5	2102.9	1946.5
141	2238.5	1841.2	1920.3	2280.8	2240.2	2225.7	2316.2	2265.2	2287.5	2300.1	2246.4	2201.2	2310.1	2300.3	2279.1	2261.6	2179.9	2168.7

**Table 8:** Acetate concentrations (mg/L) over time (days) for the different support media initially added under non-inhibiting condition.

Acetate concentration (mg/L)																		
**Sampling time (days)**	**None-1**	**None-2**	**None-3**	**Z1-1**	**Z1-2**	**Z1-3**	**Z2-1**	**Z2-2**	**Z2-3**	**AC1-1**	**AC1-2**	**AC1-3**	**AC2-1**	**AC2-2**	**AC2-3**	**X-1**	**X-2**	**X-3**
0	0	4.51	52.39	76.68	14.7	22.66	7.82	14.08	55.09	16.52	60.42	0	0	75.2	0	0	7.37	0
3	280.86	300.65	386.56	138.19	211.18	167.92	554.31	535.2	544.8	498.62	517.53	516.43	499.39	508.82	533.92	553.38	571.31	552.06
7	559.09	480.15	491.2	382.66	465.72	417.58	381.18	361.75	373.66	378.25	365.09	380.52	431.78	446.93	495.28	412.53	456.85	445.72
16	22.13	4.35	10.89	4.2	6.09	4.83	3.88	4.23	4.04	5.07	8	7.67	8.79	7.71	11.12	7.16	21.68	12.09
23	24.69	34.27	28.37	0	0	0	7.72	2.81	10.34	0	0	0	0	6.79	9.65	36.65	23.52	17.82
31	3.75	4.15	3.98	4.52	3.82	5.23	3.83	4.31	4.7	1.77	1.93	2.25	3.16	2.97	2.83	4.51	4.53	4.2

**Table 9:** Acetate concentrations (mg/L) over time (days) for the different support media initially added in presence of 19 g/L of Total Ammonia Nitrogen.

Acetate concentration (mg/L)																		
**Sampling time (days)**	**NoneN1**	**NoneN2**	**NoneN3**	**Z1N1**	**Z1N2**	**Z1N3**	**Z2N1**	**Z2N2**	**Z2N3**	**AC1N1**	**AC1N2**	**AC1N3**	**AC2N1**	**AC2N2**	**AC2N3**	**XN1**	**XN2**	**XN3**
0	0	0	0	0	0	0	0	0	0	0	0	0	0	0	0	0	0	0
3	1264.85	1304.9	1515.75	1446.4	1739.55	1540.5	1530.75	1543.1	1651.15	1233.2	975.15	1548.75	1381.85	1373.85	1158.45	1698.45	1545.85	1469.8
7	2356.2	2468.9	2504	2444.6	2631.65	2499.6	2268.35	2143.6	2210.45	2186.65	2436.25	2489.45	2449.65	2428.5	2180.4	2711.05	2795.15	2701.15
16	3007.3	3164.85	3088.9	2944.95	3121.2	3152.75	3035.3	2909.15	3079.8	3071.45	3088.9	3329.7	3171.2	2147.4	2985.55	3401.1	3219.65	3463.2
23	3949.75	3804.1	3756.3	3338.75	3307.85	3407.4	3209.8	3196.5	3241.75	3481.2	3504.95	3550.8	3468.55	3408.8	3218.2	3712.65	3518	3814.8
31	3262.9	3349.45	3311.4	2906.95	2643.95	2729.35	2445.4	2645	2520.25	3507.35	3912.1	3316.15	3544.4	3368.6	3544.95	3684.3	3419.85	3745.25
43	3331.2	3652.55	3768.1	3110.2	2653.55	2455.2	2312.8	2119.45	1901.45	3717.3	3975.55	3423.55	3441.6	3319.5	3310.1	3708.25	2880.35	3551.55
50	3064.85	3076.65	3576.7	2974.45	3048.85	2239.05	2052.75	1484.25	1143.75	3387.1	3600.6	3020.2	3237.7	3326.3	3316.85	3523.7	2946.7	3433.6
60	2625.05	2016.3	3263.25	2761.1	2455.2	2198.95	2334.1	1158.75	413.3	3200.4	2846.45	2748.5	1395.8	2144.95	1531.3	3315.1	2496.15	3121.8
85	1728.1	1222.2	1126.2	354.4	194.9	896.2	1422.4	1491.35	562.55	2431.4	3448.75	1457.55	2601.75	2950.4	2474.45	2469.3	1803.85	2238.8
106	1792.6	765.7	828.45	262.25	207.25	431.5	587.7	973.95	120.95	918.9	3030.05	0	1433.5	1423.55	1863.25	1062.15	2297.75	934.65
119	1456.7	0	0	0	0	0	0	0	0	0	707.65	0	628.95	327.9	1330.4	700.65	1112.45	1539.65

**Table 10:** Acetate concentrations (mg/L) over time (days) for the different support media initially added in presence of 1.3 g/L of phenol.

Acetate concentration (mg/L)																		
**Sampling time (days)**	**NonePhi1**	**NonePhi2**	**NonePhi3**	**Z1Phi1**	**Z1Phi2**	**Z1Phi3**	**Z2Phi1**	**Z2Phi2**	**Z2Phi3**	**AC1Phi1**	**AC1Phi2**	**AC1Phi3**	**AC2Phi1**	**AC2Phi2**	**AC2Phi3**	**XPhi1**	**XPhi2**	**XPhi3**
0	0	14.84	160.38	52.34	35.12	0	0	0	0	0	0	0	0	0	0	0	0	0
3	2767.4	2962	3186.6	2734.2	2502.6	2368.85	2602.55	2495.3	2497.4	2557.7	2476.65	2420.9	2489.85	2492.75	2442.75	2612.65	2572.35	2526.55
7	3056.45	3020.8	2959.85	2835.4	2806.05	2680	2870.65	2935.35	2944.8	2650.3	2483.8	2232.25	3062.8	3025.4	3176.1	2992.25	3004.7	2910
16	3729.3	3601.5	3719.25	3587.55	3483.45	3431.6	3360.8	3347.4	3569.65	323.9	364.5	456.6	2064.2	1995.75	2019.3	3744.9	3781.25	3870.05
23	4631.45	5275.9	4874.25	2565.4	2759.3	2370.6	2332.65	2380.45	2592.8	41.1	61.95	50	215.45	203.4	407.05	6408.05	5969.3	4774.25
31	1043.3	1793.85	2333.95	474.5	396.95	357.3	437.2	383.1	433.8	24.5	9.35	11.15	14.45	12.9	0	782.95	625.8	584.05
43	379.45	541.45	721.65	231.3	242.4	162.9	215.85	203.95	228.85	111.05	204.95	52.15	18	17.25	17.4	349.7	383.9	390.05
50	255.4	258.15	383.25	208.7	280.5	236.55	224.8	220.1	184.55	0	0	0	370.75	193.15	272.25	158.2	55.9	154.45
60	269.6	277.9	189.65	37.75	66.15	35.8	113.45	137.15	122	0	0	0	46.15	105.3	26.35	566.5	394.55	165.45
85	290.65	69.3	572.3	104.55	13.95	31.7	0	21.25	0	0	0	0	0	0	0	353.8	22.6	12.7

**Table 11:** Propionate concentrations (mg/L) over time (days) for the different support media initially added under non-inhibiting condition.

Propionate concentration (mg/L)																		
**Sampling time (days)**	**None-1**	**None-2**	**None-3**	**Z1-1**	**Z1-2**	**Z1-3**	**Z2-1**	**Z2-2**	**Z2-3**	**AC1-1**	**AC1-2**	**AC1-3**	**AC2-1**	**AC2-2**	**AC2-3**	**X-1**	**X-2**	**X-3**
0	0	0	0	0	0	0	0	0	0	0	0	0	0	0	0	0	0	0
3	124.3	119.99	128.11	84.18	112.74	107.43	186.83	187.56	171.23	191.14	198.15	194.42	177.97	180.58	181.68	191.2	196.92	191.97
7	240.42	237.53	221.7	208.92	238.05	206.85	210.18	230.05	206.31	238.94	211.09	206.63	196.09	201.51	195.96	196.77	211.96	207.84
16	264.78	262.69	267.04	235.78	250.46	248.21	254.86	251.36	265.51	229.14	231.77	232.96	235.43	246.45	241.31	278.94	273.54	270.01
23	79.95	0	43.29	0	0	0	0	0	0	0	0	0	0	0	0	10.32	13.76	21.81
31	0	0	0	0	0	0	0	0	0	0	0	0	0	0	0	0	0	0
43	0	0	0	0	0	0	0	0	0	0	0	0	0	0	0	0	0	0

**Table 12:** Propionate concentrations (mg/L) over time (days) for the different support media initially added in presence of 19 g/L of Total Ammonia Nitrogen.

Propionate concentration (mg/L)																		
**Sampling time (days)**	**NoneN1**	**NoneN2**	**NoneN3**	**Z1N1**	**Z1N2**	**Z1N3**	**Z2N1**	**Z2N2**	**Z2N3**	**AC1N1**	**AC1N2**	**AC1N3**	**AC2N1**	**AC2N2**	**AC2N3**	**XN1**	**XN2**	**XN3**
0	0	0	0	0	0	0	0	0	0	0	0	0	0	0	0	0	0	0
3	640.1	685.1	937.8	942.1	1024.7	952.4	940	953.8	1002.3	793	457.8	935.1	884.4	840.4	790.8	1010.6	960.9	907.1
7	874.2	924.5	1038.8	943.6	1001.9	958.6	918.3	853.5	910.9	760.4	659.5	853.8	868.2	907.5	784.8	1006.9	964.5	968.6
16	1021.6	1108.1	1047.9	1167.3	1145.4	1144	1101.8	1078.8	1108	926.3	772.7	1034.9	1041.4	685.4	957.2	1312.2	1113	1211.4
23	1406.8	1403.9	1232.7	0	1323.5	1367.1	1188.2	1162.3	1181.5	1115.2	986.4	838.8	1090.1	1128.7	1035.1	1391.6	1206.1	1350.4
31	1052.6	1219.7	1095.3	1118.8	1183.6	1137.9	1105.7	1112.7	1156.3	981.7	890.2	1105	1209.9	1131.3	1130.1	1344.8	1256.2	1301.3
43	1190.2	1236.8	1222.1	1324.9	1355.9	1256.1	1119.8	1250.5	1221	1065.3	929.6	1169.2	1186	1155.4	1086.8	1348.5	1161.7	1170.8
50	984.2	984.5	1270.7	1259.6	1329.3	1287	1185.7	1168.6	1265.3	984.4	873.9	1035	1100.3	1130.8	1055.5	1241.1	1114.5	1164.6
60	1079.4	0	1102	1037.1	1181.1	1131	1064.8	1225.9	1290.2	939.9	805	1016.2	931.2	1036.3	823.9	1152.1	1007.6	1026.1
85	1202	1317.7	1330.6	1318.6	1208.2	782.7	835.6	234.1	0	1113	921.8	1337.6	1239.9	1270.7	1188	1339.4	1351.4	1271.9
106	1014.9	1409.5	1332.8	0	0	0	0	0	0	1245.4	976.3	1214.7	1223.3	1193	1153	1442.6	1399.6	1332
119	0	1566.1	1240.5	0	0	0	0	0	0	925.9	1038.1	0	1344.4	1134.4	1279.8	1163.1	1365.7	1308.1

**Table 13:** Propionate concentrations (mg/L) over time (days) for the different support media initially added in presence of 1.3 g/L of phenol.

Propionate concentration (mg/L)																		
**Sampling time (days)**	**NonePhi1**	**NonePhi2**	**NonePhi3**	**Z1Phi1**	**Z1Phi2**	**Z1Phi3**	**Z2Phi1**	**Z2Phi2**	**Z2Phi3**	**AC1Phi1**	**AC1Phi2**	**AC1Phi3**	**AC2Phi1**	**AC2Phi2**	**AC2Phi3**	**XPhi1**	**XPhi2**	**XPhi3**
0	0	0	0	0	0	0	0	0	0	0	0	0	0	0	0	0	0	0
3	837.6	865.4	953	767.1	698.3	628.3	757.9	676.8	720.6	699.5	687.6	701.4	666.7	715.9	689.8	736.6	712.6	715.7
7	954.6	918.9	944.2	772.2	804.3	776.7	874.3	863.9	905	837.8	836.5	740.5	803.5	863.1	850.3	933	939.7	900.9
16	1019.5	944	984.3	851.8	874.3	777.1	936.7	927	985.3	1045.7	1251	1206.3	1168.4	1152.7	1090.8	760.1	784.6	799.3
23	1046.6	1002.7	942.1	1050.3	1059	973.3	1094.4	1040.6	1007.4	0	0	0	845.9	1199.7	1248.5	1324.9	1237.2	1068.2
31	1236.8	1173.8	1224.7	1168.4	1180	1109	1197.2	1196.3	1198.7	0	0	0	0	0	0	1182.9	1153.4	1162.3
43	1352.1	1270.3	1291.7	1120.7	1218.6	255.2	1068.1	1326.9	1255.7	1281.5	0	0	0	0	0	1335.9	1315.1	1284.2
50	1111.6	1369.1	1480.6	0	77.2	0	548.2	710.6	555.5	0	0	0	0	0	0	1323.4	1097	690.5
60	198.2	445.5	1088.9	0	0	0	0	0	0	0	0	0	0	0	0	0	186.6	0
85	0	0	0	0	0	0	0	0	0	0	0	0	0	0	0	0	0	0

**Table 14:** Butyrate concentrations (mg/L) over time (days) for the different support media initially added under non-inhibiting condition.

Butyrate concentration (mg/L)																		
**Sampling time (days)**	**None-1**	**None-2**	**None-3**	**Z1-1**	**Z1-2**	**Z1-3**	**Z2-1**	**Z2-2**	**Z2-3**	**AC1-1**	**AC1-2**	**AC1-3**	**AC2-1**	**AC2-2**	**AC2-3**	**X-1**	**X-2**	**X-3**
0	0	0	0	0	0	0	0	0	0	0	0	0	0	0	0	0	0	0
3	106.3	124.4	197.6	35.6	210.6	188.4	268.5	261	278.6	204.7	206.7	207.6	243.2	245.9	237	269.2	261.1	259.6
7	210	189.6	195.6	109.7	158.9	136.8	134.9	105.4	137.3	91.8	100.9	98.1	115.7	126.2	156	139.9	156.8	149.1
16	0	0	0	0	0	0	0	0	0	0	0	0	0	0	0	0	0	0
23	0	0	0	0	0	0	0	0	0	0	0	0	0	0	0	0	0	0
31	0	0	0	0	0	0	0	0	0	0	0	0	0	0	0	0	0	0

**Table 15:** Butyrate concentrations (mg/L) over time (days) for the different support media initially added in presence of 19 g/L of Total Ammonia Nitrogen.

Butyrate concentration (mg/L)																		
**Sampling time (days)**	**NoneN1**	**NoneN2**	**NoneN3**	**Z1N1**	**Z1N2**	**Z1N3**	**Z2N1**	**Z2N2**	**Z2N3**	**AC1N1**	**AC1N2**	**AC1N3**	**AC2N1**	**AC2N2**	**AC2N3**	**XN1**	**XN2**	**XN3**
0	0	0	0	0	0	0	0	0	0	0	0	0	0	0	0	0	0	0
3	0	0	365.4	358	295.5	294.8	328.5	338.3	345.2	317.8	330.3	308.8	298.5	264.5	311.2	351.3	346.8	344.2
7	0	433.9	401.3	402	307.4	308.5	341.1	327.8	369.2	273.1	407.3	275.6	280.4	279.5	319.4	374.1	340.3	344.4
16	0	0	0	0	0	0	0	0	0	0	0	0	0	0	0	0	0	0
23	0	0	0	0	0	0	0	0	0	0	0	0	0	0	0	0	0	0
31	0	0	0	0	0	0	0	0	0	0	0	0	0	0	0	0	0	0
43	0	0	0	0	0	0	0	0	0	0	0	0	0	0	0	0	0	0
50	0	0	0	0	0	0	0	0	0	0	0	0	0	0	0	0	0	0
60	0	0	0	0	0	0	0	0	0	0	0	0	0	0	0	0	0	0
85	0	0	0	0	0	0	0	0	0	0	0	0	0	0	0	0	0	0
106	0	0	0	0	0	0	0	0	0	0	0	0	0	0	0	0	0	0
119	0	0	0	0	0	0	0	0	0	0	0	0	0	0	0	0	0	0

**Table 16:** Butyrate concentrations (mg/L) over time (days) for the different support media initially added in presence of 1.3 g/L of phenol.

Butyrate concentration (mg/L)																		
**Sampling time (days)**	**NonePhi1**	**NonePhi2**	**NonePhi3**	**Z1Phi1**	**Z1Phi2**	**Z1Phi3**	**Z2Phi1**	**Z2Phi2**	**Z2Phi3**	**AC1Phi1**	**AC1Phi2**	**AC1Phi3**	**AC2Phi1**	**AC2Phi2**	**AC2Phi3**	**XPhi1**	**XPhi2**	**XPhi3**
0	0	0	0	0	0	0	0	0	0	0	0	0	0	0	0	0	0	0
3	1590.3	1719.9	1846.8	1714.15	1541.6	1483.65	1493.85	1445.95	1439.7	1410.3	1383	1320	1489.65	1492.5	1460.25	1484.55	1436.9	1383.65
7	1365.1	1407.1	1396.4	1371.8	1366.4	1312.9	1344.1	1350.75	1370.5	1097.65	1073.15	1004.3	1274.95	1306.95	1302.8	1448.35	1446.45	1348.45
16	1109.5	1028.5	1006.2	921.7	982.65	693.1	1026.7	1092.45	1031.3	0	0	0	0	0	0	1257.85	1123.1	977.4
23	256.75	315.75	337.15	0	0	0	0	0	0	0	0	0	0	0	0	417.3	0	0
31	0	0	0	0	0	0	0	0	0	0	0	0	0	0	0	0	0	0
43	0	0	0	0	0	0	0	0	0	0	0	0	0	0	0	0	0	0
50	0	0	0	0	0	0	0	0	0	0	0	0	0	0	0	0	0	0
60	0	0	0	0	0	0	0	0	0	0	0	0	0	0	0	0	0	0
85	0	0	0	0	0	0	0	0	0	0	0	0	0	0	0	0	0	0

## 2 Experimental design, materials and methods

### 2.1 Experimental design and sampling

54 anaerobic batch bioreactors were initially seeded with 20 g of centrifuged methanogenic sludge as inoculum and supplemented with 50 g of mashed biowaste as substrate corresponding to an initial organic loading of 10 g COD/g COD. A total of 5 support media (2 different zeolites, 2 different activated carbons and one type of chitosan) were tested. One triplicate of bioreactors without support was also implemented as control. In a first set of 18 bioreactors, NH_4_Cl (99.998%, Sigma Aldrich) was added in order to reach 19 g/L of total ammonia nitrogen. In the second set of 18 bioreactors, phenol (99%, ACROS Organics) was added in order to reach 1.5 g/L. The last set of 18 bioreactors was not supplemented with inhibitor. Time zero (T0) samples were taken and all reactors were incubated without agitation, in the dark, at 35 °C. Liquid samples (2 mL) were periodically taken through the septum and centrifuged at 10,000*g* for 10 min. Pellets were separated from the supernatant and stored at − 20 °C.

### 2.2 DNA extraction, amplification and sequencing

Total DNA was extracted from the pellet using PowersoilTM DNA isolation kit (Mobio Laboratories Inc. Carlsbad) according to the manufacturer׳s instructions. DNA extracts were used for the amplification of the bacterial and archaeal hypervariable region V4–V5 of the 16S rRNA genes with the primers 515F (5′-GTGYCAGCMGCCGCGGTA-3′) and 928R (5′-CCCCGYCAATTCMTTTRAGT-3′) as described in [1], [2]. Sequencing was performed on Ion Torrent Personal Genome Machine using Ion 316 chip and the Ion PGM Sequencing 400 Kit.

### 2.3 Sequence read processing

PGM software filtered out low quality and polyclonal sequence reads, and quality filtered data was exported as FastQ file.
